# Low-Energy Electron Scattering from 2‑Butyne: Elastic and Electronic Excitation Processes

**DOI:** 10.1021/acsomega.6c05377

**Published:** 2026-08-17

**Authors:** Murilo O. Silva, Luiz V. S. Dalagnol, Giseli M. Moreira, Romarly F. da Costa, Márcio. H. F. Bettega

**Affiliations:** † Departamento de Física, Universidade Federal do Paraná, Caixa Postal 81531-980 Curitiba 19044, Paraná, Brazil; ‡ Laboratório de Simulações Computacionais em Moléculas, Agregados e Sólidos, Núcleo de Modelagem e Computação Científica, Centro Interdisciplinar de Ciência, Tecnologia e Inovação, Universidade Federal Do Paraná, Curitiba 81531-980, Paraná, Brazil; § Centro de Ciências Naturais e Humanas, 74362 Universidade Federal Do ABC, Santo André, São Paulo 09210-580, Brazil

## Abstract

We report integral and differential cross sections for elastic and electronically inelastic electron scattering from the 2-butyne molecule. We considered excitations from the ground state to the 1^3^ *B*_2_, 1^3^ *A*_2_, 2^3^ *B*_2_, 2^3^ *A*_2_, 1^1^ *A*_2_, 2^1^ *A*_2_, 1^1^ *B*_2_, 1^1^ *A*_1_ and 1^1^ *B*_1_ excited states. The scattering amplitudes were computed using the Schwinger multichannel method implemented with norm-conserving pseudopotentials for incident electron energies from 0.01 eV up to 50 eV. The calculations were performed within a minimal orbital basis for single–configuration interaction strategy, at the close-coupling level including up to 247 open channels. In addition, total ionization cross sections were obtained using the Binary-Encounter-Bethe model. By combining the ionization cross sections with the present elastic and electronically inelastic results, we provide an estimate for the total cross section.

## Graphical abstract

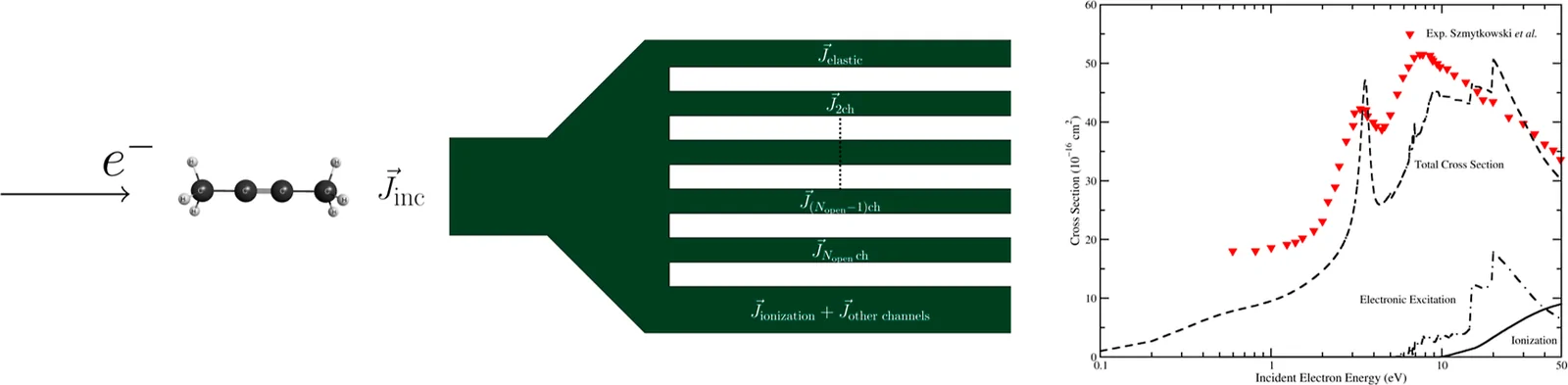

## 1 Introduction

The landmark study by Boudaiïffa et al.^1^ established that low-energy secondary electrons induce Deoxyribonucleic acid (DNA) strand breaks via their capture into unoccupied molecular orbitals. Such transient negative ions (also called resonances) provide a critical pathway for DNA degradation, prompting further investigation into complex biological subunits.^2–5^ Understanding these interactions is essential for mapping radiation-induced tissue damage and the chemical dynamics of DNA constituent molecules.^6,7^ In the technological area, particular interest is devoted to the so-called cold plasmas, which are widely used in the development of electronic devices and materials with modified properties, mainly by layer deposition, in addition to controlled corrosion cleaning and surface decontamination.^8–12^ To obtain a plasma, a small amount of a gaseous species is submitted to alternating electric and magnetic fields, leading to its partial ionization. During this process, the released electrons, upon colliding with the molecules present in the chamber, produce secondary species.^13,14^ For the mathematical modeling of such plasmas, cross section data for electron collisions with plasma species are essential.^15^

Based on the applications mentioned above, we decide to conduct a study involving the 2-butyne (C_4_H_6_, see Figure 1) molecule, which is an alkyne hydrocarbon, characterized as a colorless, flammable, and corrosive liquid. Structurally, it is derived from ethyne by substitution of both hydrogen atoms with methyl groups. This compound is widely employed in catalytic organic synthesis and in the production of halogenated derivatives (such as halothanes) via reaction with basic alcoholic solutions.^17^ Furthermore, it acts as a precursor in the formation of tetrahydrofuran (THF)-free metallacyclobutenes.^18,19^

**Figure 1:**
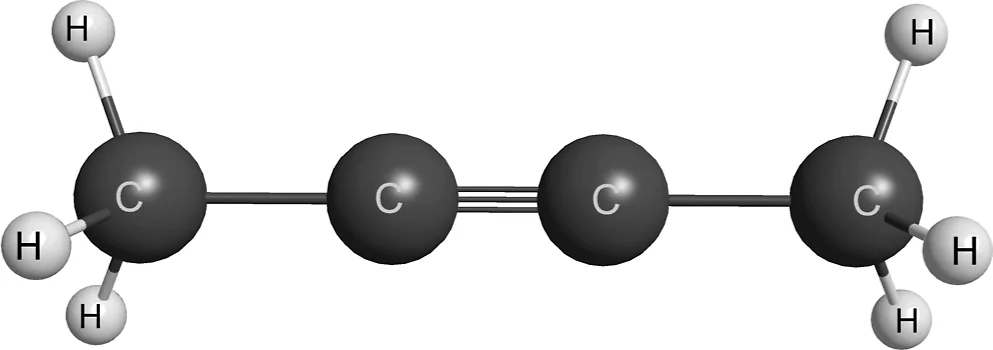
Ball-and-stick model of the 2-butyne (2-C_4_H_6_) molecule generated with MacMolPlt.^16^

Regarding previous studies available in the literature for the 2-butyne, we highlight the theoretical studies by Lopes et al.^20^ and Khakoo et al.,^21^ both of which employed the Schwinger multichannel method and considered only the elastic channel. From the experimental perspective, Khakoo et al.^21^ used electron energy-loss spectroscopy (EELS) to obtain differential cross sections. Additionally, Szmytkowski et al.^22^ measured total cross sections for 2-butyne using the linear electron-transmission method, while Dance & Walker^23^ used an electron trap apparatus with a trochoidal monochromator to measure threshold electron energy-loss spectra for several simple alkynes, identifying low-lying triplet states and resonances. It is important to mention that, to the best of our knowledge, no data on electronically inelastic cross sections for 2-butyne are available in the literature.

In the present study, we investigate the elastic and electronically inelastic electron scattering processes from the 2-butyne molecule. The scattering calculations were carried out using the minimal orbital basis for single–configuration interaction strategy^24^ and considered up to 247 open channels. We used the Schwinger multichannel method^25–27^ in its version that makes the use of norm-conserving pseudopotentials^28,29^ of Bachelet, Hamann, and Schlüter (BHS).^30^ We report integral, differential, and total cross sections for 2-butyne at electron impact energies up to 50 eV. Additionally, we employed the Binary-Encounter-Bethe (BEB) model^31,32^ to compute the ionization cross section.

This manuscript is organized as follows: In Section 2, we present a brief summary of the most important aspects of the Schwinger multichannel method. Section 3 provides an explanation of the computational details of the present calculations. Our results and discussion are presented in Section 4. We close the paper presenting our conclusions in Section 5.

## 2 Theory

In our calculations, we employed the Schwinger MultiChannel (SMC) method^25–27^ implemented with norm-conserving pseudopotentials^28,29^ (PP) proposed by Bachelet, Hamann, and Schlüter (BHS),^30^ to obtain elastic and electronically inelastic cross sections. The SMC method is a variational method to the scattering amplitude which accounts for fundamental effects in the electron–molecule interaction process, such as the exchange between the continuum electron and the target electrons, the polarization effect induced by the electric field of the incident electron—which distorts the target’s electronic cloud—and the multichannel coupling, in which multiple scattering channels are open. In particular, the explicit inclusion of additional physical channels provides alternative pathways for the scattering flux and prevents the artificial overestimation of cross sections associated with an incomplete multichannel expansion. Since the SMC method has been thoroughly reviewed in ref 27 we present here only the aspects relevant to the present calculations. Within the SMC framework, the scattering amplitude can be written as

1 $$f^{SMC}\left({\mathrm{k⃗}}_{f},{\mathrm{k⃗}}_{i}\right)=-\frac{1}{2\pi }\sum_{\mu =1}^{N_{CSF}}\sum_{\nu =1}^{N_{CSF}}\langle S_{{\mathrm{k⃗}}_{f}}|V|\chi_{\mu }\rangle {\left(d^{-1}\right)}_{\mu \nu }\langle \chi_{\nu }|V|S_{{\mathrm{k⃗}}_{i}}\rangle$$

where *N*_CSF_ is the total number of configuration-state functions (CSFs), and

2 $$d_{\mu \nu }=\langle \chi_{\mu }|A^{\left(+\right)}|\chi_{\nu }\rangle$$

are the matrix elements of the denominator operator. Such matrix elements involve the operator *A* ^(+)^ given by

3 $$A^{\left(+\right)}=\frac{Ĥ}{N+1}-\frac{PĤ+ĤP}{2}+\frac{PV+VP}{2}-VG_{P}^{\left(+\right)}V$$

In these expressions, $|S_{{\mathrm{k⃗}}_{i\left(f\right)}}\rangle$ denotes an eigenfunction of the unperturbed Hamiltonian *H*_0_ = *H*_*N*_ + *T*_*N*+1_, constructed as the direct product of a target electronic state and a plane wave. The vector ${\mathrm{k⃗}}_{i\left(f\right)}$ corresponds to the momentum of the incoming (outgoing) electron. In this partition, *H*_*N*_ describes the internal Hamiltonian of the *N*-electron target, while *T*_*N*+1_ represents the kinetic energy operator associated with the projectile electron. The operator *V* accounts for the interaction between the incident electron and both the electrons and nuclei of the target. Furthermore, we define *Ĥ* = *E-H*, where *E* is the total scattering energy and *H* is the full (*N* + 1)-electron Hamiltonian within the fixed-nuclei approximation. The quantity *G*_*P*_^(+)^ = *PG_0_^(+)^* corresponds to the free Green’s operator projected onto the subspace *P*, which spans the open channels of the target and is defined by

4 $$P=\sum_{l=1}^{N_{open}}|\Phi_{l}^{s}\rangle \langle \Phi_{l}^{s}|$$

where $|\Phi_{l}^{s}\rangle$ denotes either the ground state ( l = 1) or an electronically excited state ( l > 1) of the target molecule. The integer *N*_open_ gives the number of energetically accessible channels included in the calculation, which increases as the incident electron energy becomes larger.

The set ${\left\{|\chi_{\mu }\rangle \right\}}_{\mu =1}^{N_{CSF}}$ defines a basis of (*N* + 1)-electron Slater determinants, or configuration state functions (CSFs), built from spin-adapted combinations of target electronic states and one-electron scattering orbitals. These basis functions are expressed as

5 $$|\chi_{\mu \nu }\rangle =A_{N+1}\left[|\Phi_{\mu }^{s}\rangle ⊗|\varphi_{\nu }\rangle \right]$$

where $A_{N+1}$ denotes the *N* + 1-antisymmetrization operator. The ket |Φ_μ_^s^⟩ corresponds to an *N*-electron state of the molecular target. In particular, |Φ_1_^0^⟩ represents the Hartree–Fock (HF) ground state, while the excited states |Φ_μ_^s^⟩ (μ ≥ 2) are generated through single excitations from the occupied valence orbitals of the reference state into a selected set of virtual orbitals, with total spin *s* (*s* = 0 for singlet and *s* = 1 for triplet). The ket |φ_ν_⟩ describes the scattering orbital.

The ionization channel is not yet implemented in the SMC method. Therefore, to derive a total ionization cross section (TICS) we employed the Binary-Encounter-Bethe (BEB) model.^31,32^

In this model, the ionization cross section of the *i*-th molecular orbital is given by

6 $$\sigma_{i}\left(t_{i}\right)=4\pi a_{0}^{2}\left\{\frac{N_{i}}{1+t_{i}+u_{i}}{\left(\frac{R}{B_{i}}\right)}^{2}\left[\frac{\ln t_{i}}{2}\left(1-\frac{1}{t_{i}^{2}}\right)+1-\frac{1}{t_{i}}-\frac{\ln t_{i}}{1+t_{i}}\right]\right\}$$

where *a*_0_ is the Bohr radius (≈0.5292 Å), *N*_*i*_ is the occupation number of the ionizing orbital, and *R* is the Rydberg energy (≈13.61 eV). The remaining parameters, namely, *t*_*i*_ = *E*/*B*_*i*_, and *u*_*i*_ = *U*_*i*_/*B*_*i*_, are obtained from ground state electronic structure calculations, since *B*_*i*_ is the bound state binding energy of the ionizing orbital, *U*_*i*_ is the orbitals’ average kinetic energy and *E* is the incident electron energy. From the partial ionization cross sections one obtains the TICS through

7 $$\sigma_{BEB}=\sum_{i=1}^{N_{occ}}\sigma_{i}\left(t_{i}\right)$$

where *N*_occ_ is the total number of occupied molecular orbitals. All quantities entering the BEB formalism, were obtained from canonical HF molecular orbitals calculated at the equilibrium geometry of the ground electronic state using the aug-cc-pVDZ basis set, as implemented in the GAMESS^33,34^ package. It is important to note that the ionization channel does not compete for the flux that defines the elastic and electronically inelastic cross sections obtained with the SMC method, nor do these channels compete for the flux that defines the ionization cross section.

## 3 Computational Details

The 2-butyne molecule belongs to the D_3*h*_ point group; however, all calculations were carried out in the reduced C_2*v*_ symmetry. The correlation between the irreducible representations of these two groups is presented in Table 1.

**Table 1:** Relation between the Irreducible Representations of D_3*h*_ and C_2*v*_ Point Groups^35,36^

D_3h_	C_2v_
*A*_1_^′^	*A*_1_
*A*_2_^′^	*B*_1_
*E*′	*A*_1_ ⊕ *B*_1_
*A*_1_^″^	*A*_2_
*A*_2_^″^	*B*_2_
*E*″	*A*_2_ ⊕ *B*_2_

The ground state geometry of the molecule was optimized using second-order Møller–Plesset perturbation theory (MP2) with the 6–31G­(1*d*) basis set, as implemented in the GAMESS^33,34^ computational package. For both the bound-state and scattering calculations, the pseudopotentials of Bachelet et al.^30^ were employed to represent the nuclei and core electrons of the carbon atoms. The Cartesian Gaussian functions used for the carbon atoms consisted of a 5*s*5*p*3*d* set and were described elsewhere.^37^ For the hydrogen atoms, the 4*s*/3*s* basis set of Dunning Jr.^38^ was employed, with the addition of a *p*-function with exponent 0.75.

As mentioned before, the molecular target ground state is described using the HF method, while the excited states are treated using the Minimal Orbital Basis for Single-Configuration Interaction (MOB–SCI)^24^ strategy. In general terms, the MOB–SCI strategy consists of selecting the hole–particle (HP) pairs that contribute most significantly to the description of the electronic states of interest. This selection is performed based on a Full Single-Configuration Interaction (FSCI) calculation, from which the dominant pairs responsible for describing the excited states are identified. The purpose of this strategy is to reduce the configurational space while retaining the most relevant electronic contributions, thereby making it feasible to include the most important excited states in electron scattering calculations while avoiding the prohibitive computational cost associated with including all excited states. To perform the scattering calculations, the following procedure was adopted: (i) first, improved virtual orbitals (IVOs)^39^ were used to represent the particle and scattering orbitals employed in both bound-state and scattering calculations; (ii) next, a FSCI calculation was carried out, which yielded 1947 singly excited Slater Determinants (i.e., 1947 HP pairs). This corresponds to a total of 3894 electronically excited states, comprising 1947 singlet and 1947 triplet states; (iii) from this set of 3894 reference states, the 200 lowest-energy states were selected. These states were then described using 123 HP pairs to construct the active space of the MOB–SCI strategy. This selection ensured that the excitation energies were reproduced with at least 90% agreement relative to the FSCI results.

Table 2 presents the first 16 (11 according D_3*h*_ point group) excited states obtained using the Full Single–Configuration Interaction with pseudopotentials (FSCI–PP) method, in comparison with results from the MOB–SCI strategy.

**Table 2:** Vertical Excitation Energies (in eV) for the First 38 Excited Electronic Singlet and Triplet States Obtained from FSCI–PP, MOB–SCI, FSCI–AE, TD–DFT, EOM–CCSD Calculations

state (C_*2v*_)	state (D_*3h*_)	FSCI–PP	MOB–SCI	FSCI–AE	TD–DFT	EOM–CCSD	ref 52	ref 53	ref 54
${\mathrm{X̃}}^{1}A_{1}$	$\mathrm{X̃}{}^{1}A_{1}^{\prime }$								
1^3^ *B*_2_	1^3^ *A*_2_^″^	4.541	5.051	4.687	4.774	5.482	5.671		
1^3^ *A*_2_	1^3^ *E*″	5.509	5.762	5.619	5.546	6.470	6.248		
2^3^ *B*_2_									
2^3^ *A*_2_	1^3^ *A*_1_^″^	6.352	6.452	6.442	6.404	7.136	6.515		
1^1^ *A*_2_	1^1^ *A*_1_^″^	6.353	^a^6.453	6.444	6.426	7.191	7.217		7.967
1^3^ *A*_1_	1^3^ *E*′	6.641	6.717	6.556	5.904	6.496	7.478		
1^3^ *B*_1_									
2^1^ *A*_2_	1^1^ *E*″	6.692	6.794	6.768	6.475	7.358	7.605		8.098
1^1^ *B*_2_									
1^1^ *A*_1_	1^1^ *E*′	6.899	6.939	6.784	5.997	6.605	7.858	6.806	8.010
1^1^ *B*_1_									
4^1^ *A*_2_	3^1^ *E*″	9.738	9.809	8.757	7.735	8.558	8.732		8.731
3^1^ *B*_2_									
17^1^ *A*_1_	6^1^ *A*_1_^′^	14.769	14.996	13.700	11.991		11.531		
30^1^ *A*_2_	28^1^ *A*_1_^″^		19.999						
31^1^ *B*_2_	28^1^ *A*_2_^″^		21.068						

a This state is accidentally degenerated with the 2^3^ *A*_2_ (1^3^ *A*_1_^″^) in MOB–SCI and FSCI levels of theory.^55^

Due to the scarcity of literature data for the excited states of this system, additional calculations were also performed for comparison purposes using Full Single-Configuration Interaction with all electrons (FSCI–AE) of the molecular target, as well as Time–Dependent Density–Functional Theory^40,41^ (TD–DFT) with the PBE0 functional^42–46^ and aug-cc-pVDZ basis set,^47^ and the Equation-of-Motion Coupled–Cluster with Singles and Doubles^48–51^ (EOM–CCSD) with the aug-cc-pVDZ basis set,^47^ both of which are well established in the literature. It is observed that there is excellent agreement between the reference results obtained using the FSCI-PP method and the spectrum generated by the MOB-SCI strategy. Due to the reduction of the point group, degenerate states are also present. Furthermore, our results show excellent consistency with the other methods employed to calculate the spectrum of excited states.

In Figure 2, we present an illustrative representation for the 246 vertical excitation energies obtained using the MOB-SCI strategy, along with the various schemes employed to incorporate multichannel coupling in the scattering calculations. The thresholds for each channel-coupling scheme are indicated by dashed red lines, corresponding to the 2ch, 4ch, 6ch, 8ch, 10ch, 12ch, 32ch, 143ch, 244ch, and 247ch levels of approximation.

**Figure 2:**
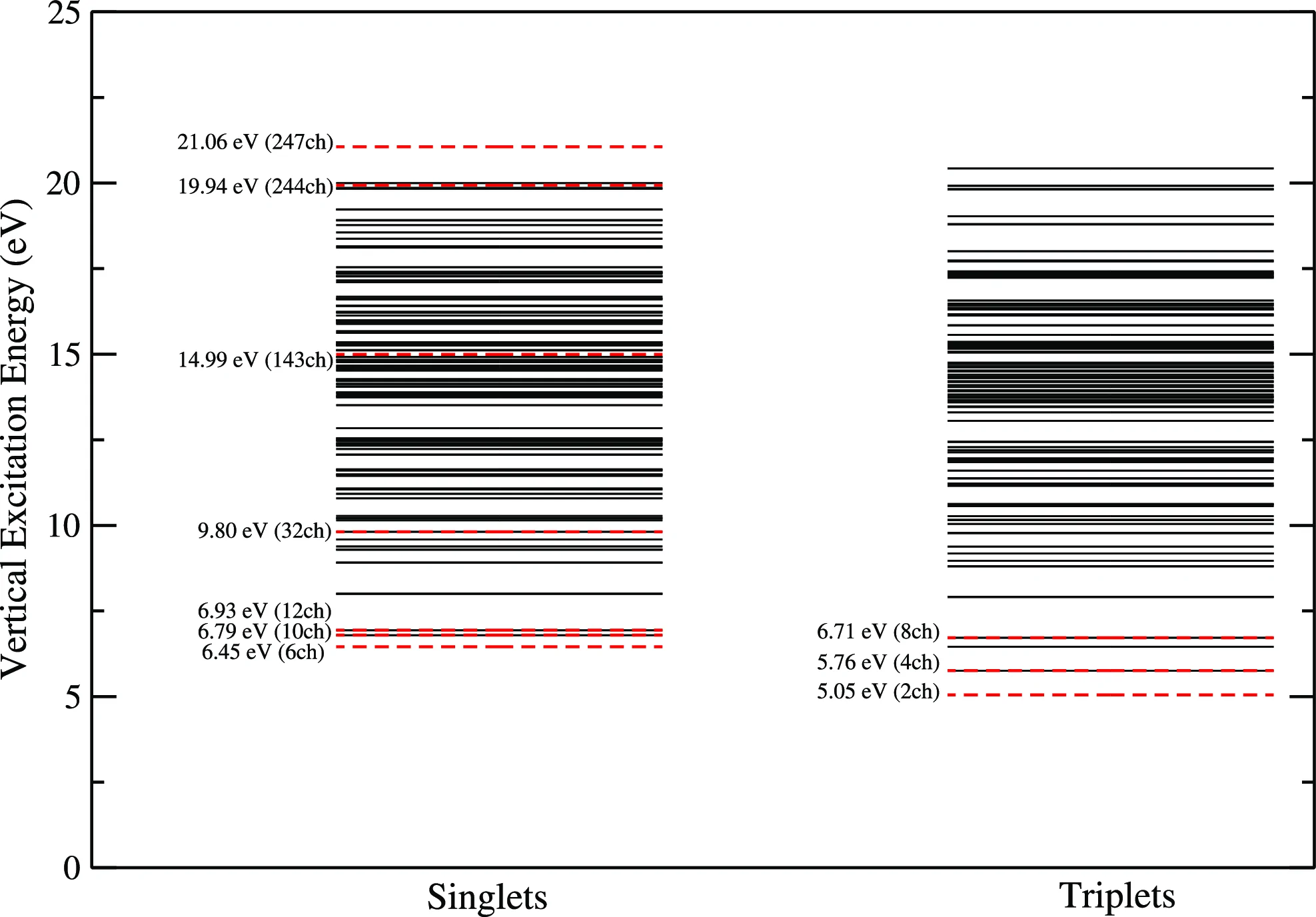
Schematic representation of the vertical excitation energies (in eV) of the 246 electronically excited states of 2-butyne obtained with the MOB-SCI calculation and the different levels of channel coupling employed in the present scattering calculations performed by means of the SMC method.

As mentioned above, 123 HP pairs were used to compose the active space in the MOB-SCI strategy. These same pairs were also employed to construct the CSF space, capturing the polarization effect of the molecular target. The number of CSFs obtained for each irreducible representation was: 10473 for *A*_1_, 10233 for *A*_2_, 10232 for *B*_1_, and 10480 for *B*_2_.

## 4 Results and Discussion

### 4.1 Elastic Scattering Resonances

In Figure 3, we present the elastic integral cross section (ICS) for electron scattering by the 2-butyne molecule, for impact energies between 0.01 and 50 eV, considering 1 to 247 energetically accessible channels. Our cross sections are compared with the theoretical results of Lopes et al.^20^ and Khakoo et al.,^21^ both obtained using the SMC method and considering only the elastic channel as open. In the work of Lopes et al.,^20^ only the static-exchange approximation is employed, in which polarization effects are not included. Khakoo et al.,^21^ in turn, included polarization effects, but adopting a polarization criterion different from that used in the present work. The comparison also includes the experimental data reported by Khakoo et al.^21^ We identified a resonant structure at 3.58 eV (π*), associated with the *A*_2_ and *B*_2_ (*E*″ of D_3*h*_) symmetries and one large structure around 8–8.50 eV. Lopes et al.^20^ reported an analogous structure at a higher energy (5.50 eV), which is consistent with the absence of polarization effects in their theoretical treatment. In turn, Khakoo et al.^21^ performed a dedicated theoretical study of the resonant states of this system and identified a structure at 3.60 eV, originating from the same symmetry as observed in the present work, in excellent agreement with our results, as the same for the large structure. Furthermore, the authors showed that the resonant orbital is localized along the C≡C triple bond and is degenerate, which follows from the fact that the molecule belongs to the D_3*h*_ point group, while the calculations were carried out in the C_2*v*_ subgroup, where this degeneracy is formally lifted. Szmytkowski et al.,^22^ and Dance & Walker^23^ also experimentally identified the resonance centered at 3.60 eV, with our results being in excellent agreement with the findings reported by both authors.

**Figure 3:**
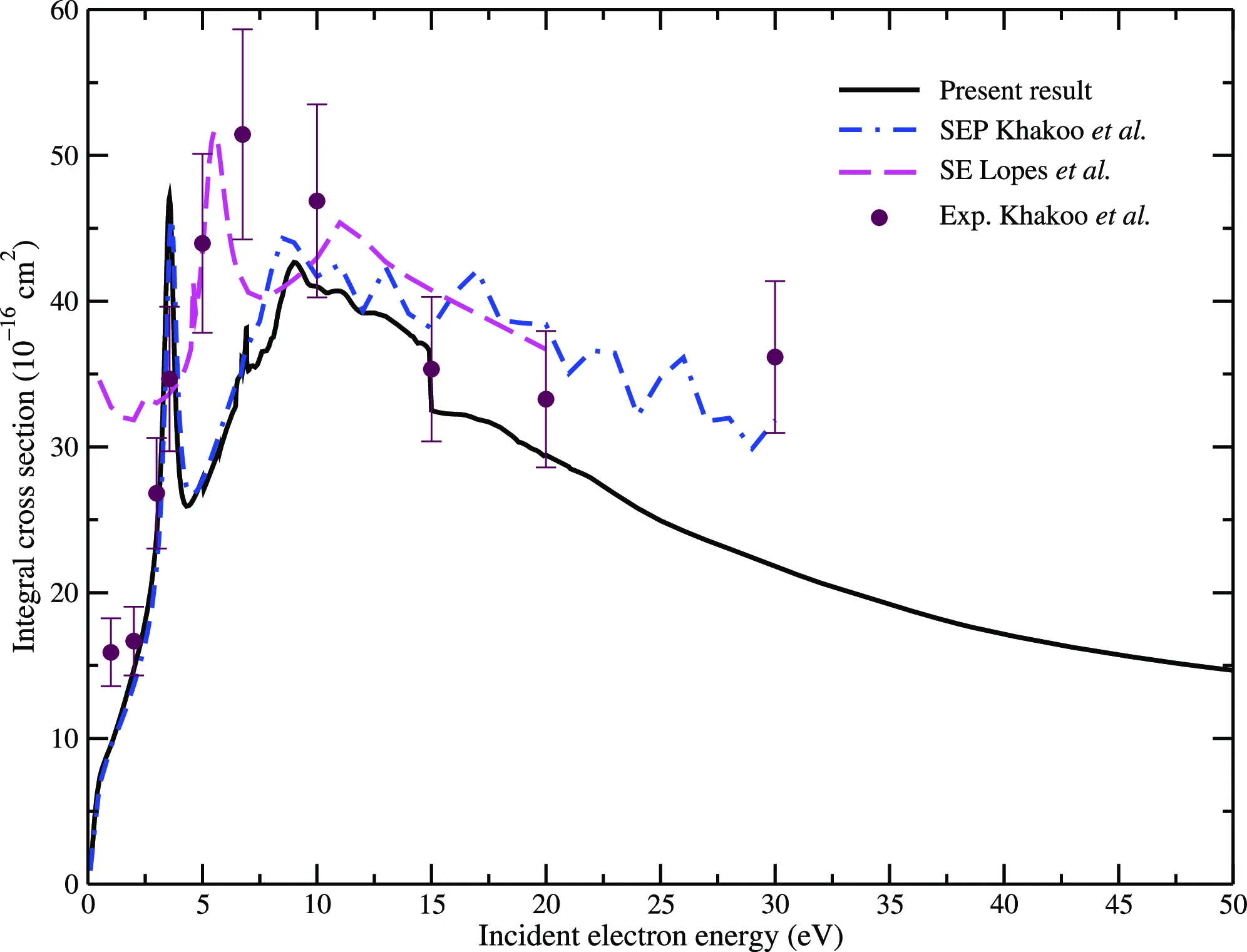
Elastic integral cross section for elastic electron scattering by 2-butyne molecule. Solid black line, present result; dashed-dotted blue line, SMC results obtained by Khakoo et al.;^21^ dashed magenta line, SMC results obtained by Lopes et al.;^20^ maroon circles, experimental measurements performed by Khakoo et al.^21^

### 4.2 Elastic Scattering Cross Sections

Regarding to the behavior of the elastic ICS (Figure 3), the results of Lopes et al.^20^ exhibit a greater magnitude than ours, which is consistent with the absence of polarization effects in their description and with the fact that they consider only the elastic channel as open in their calculations. In contrast, when compared with the theoretical results of Khakoo et al.,^21^ obtained using the SMC method, an excellent agreement for energies up to 7 eV is observed. Above 7 eV, our cross section shows a reduction in magnitude, which becomes more pronounced from 15 eV onward. This behavior arises because, as the incident electron energy increases, other inelastic channels start to compete for the scattering flux, as additional channels become energetically accessible. In contrast, the calculations of Khakoo et al.^21^ exclusively considered the elastic channel, which leads to a larger elastic cross section at higher energies. With regard to the experimental results of Khakoo et al.,^21^ we observed good agreement over the analyzed energy range, with our values lying within the experimental error bars. Exceptions occur at 5, 6.75, and 30 eV, where the experimental integral cross sections exhibit larger magnitudes than ours. Besides the uncertainties associated with the extrapolation of the differential cross sections (DCS) to 0° and 180°, required for the numerical evaluation of the integral cross sections, the finite experimental energy resolution (40–60 meV fwhm) and other experimental uncertainties, including those related to the angular resolution of the measurements, may also contribute to the remaining discrepancies, particularly in regions where the cross section changes rapidly with energy or scattering angle.

In the paper by Khakoo et al.,^21^ based on the decrease of the cross section as the impact energy tends to zero, the authors investigated and identified the presence of a Ramsauer–Townsend minimum. This phenomenon corresponds to the point at which the *s*-wave cross section vanishes and a phase inversion occurs in the *s*-wave eigenphase. In their theoretical study, this effect was identified at 0.08 eV. In Figure 4, we present the *s*-wave cross section (top frame) and the *s*-wave eigenphase (bottom frame) for elastic electron scattering from 2-butyne, compared to the results obtained by Khakoo et al.^21^ We observe that the *s*-wave cross section vanishes at 0.14 eV and that, at this same energy, the eigenphase crosses zero and changes sign, from positive to negative, thereby unequivocally characterizing the signature of a Ramsauer–Townsend minimum in our results, in good agreement with the authors’ findings.^21^

**Figure 4:**
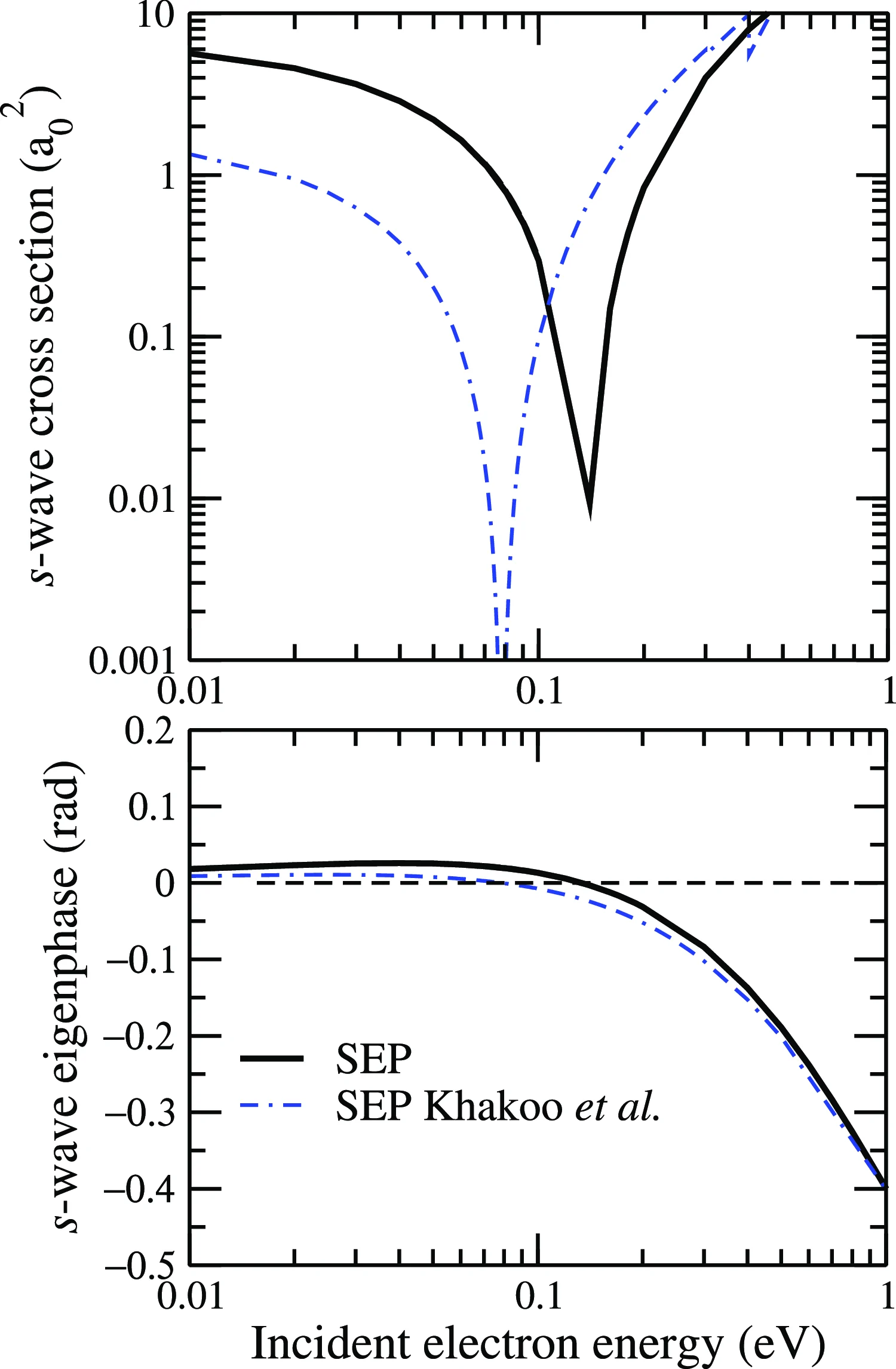
*s*-save cross section (top frame) and its eigenphase (bottom frame) for elastic electron scattering by the 2-butyne molecule. Solid black line, present SMC results; dashed-dotted blue line, SMC results by Khakoo et al.^21^

The DCSs for elastic scattering from the 2-butyne molecule at energies of 1, 2, 3, 5, 6.75, 10, 15, 20, and 30 eV are presented in Figures 5 and 6. Our results are compared with the theoretical and experimental data of Khakoo et al.^21^ With respect to the authors’ theoretical results, we observe good agreement at 1 eV. At 2 eV, there is agreement in the angular behavior of the DCS up to 90°, but with a difference in magnitude; for angles above this value, a more significant disagreement between the results is observed. For energies between 3 and 6.75 eV, good agreement is observed both in the angular behavior and in the overall shape of the distributions, with only small differences in the magnitude of the cross sections. In this energy range, however, additional channels become energetically accessible, as illustrated in the schematic representation shown in Figure 2. Specifically, the calculations successively include 2, 4, 6, and 8 channels. The opening of these new channels may give rise to threshold effects, which can influence the magnitude of the DCSs. This effect may be responsible for the small differences observed in the magnitude of the cross sections within this energy range. This difference becomes more pronounced at 10 eV, which is attributed to the larger number of open channels considered in our calculations, leading to a redistribution of the scattering flux among the accessible channels. For energies of 15, 20, and 30 eV, we observe a significant difference in magnitude relative to the theoretical results of Khakoo et al.,^21^ which was already expected, since the calculations of these authors consider only the elastic channel. In this energy range, additional channels become accessible to the molecular target, which leads to a significant reduction in the magnitude of the cross section, due to competition between the elastic and electronically inelastic channels for the cross section flux. Regarding the comparison with the experimental data of Khakoo et al.,^21^ we observe good agreement at 15 eV. At 20 eV, the authors’ results lie slightly above ours, whereas at 30 eV our results once again show satisfactory agreement with their data, despite a small difference in magnitude, which may contribute to the larger discrepancy observed in the corresponding ICS.

**Figure 5:**
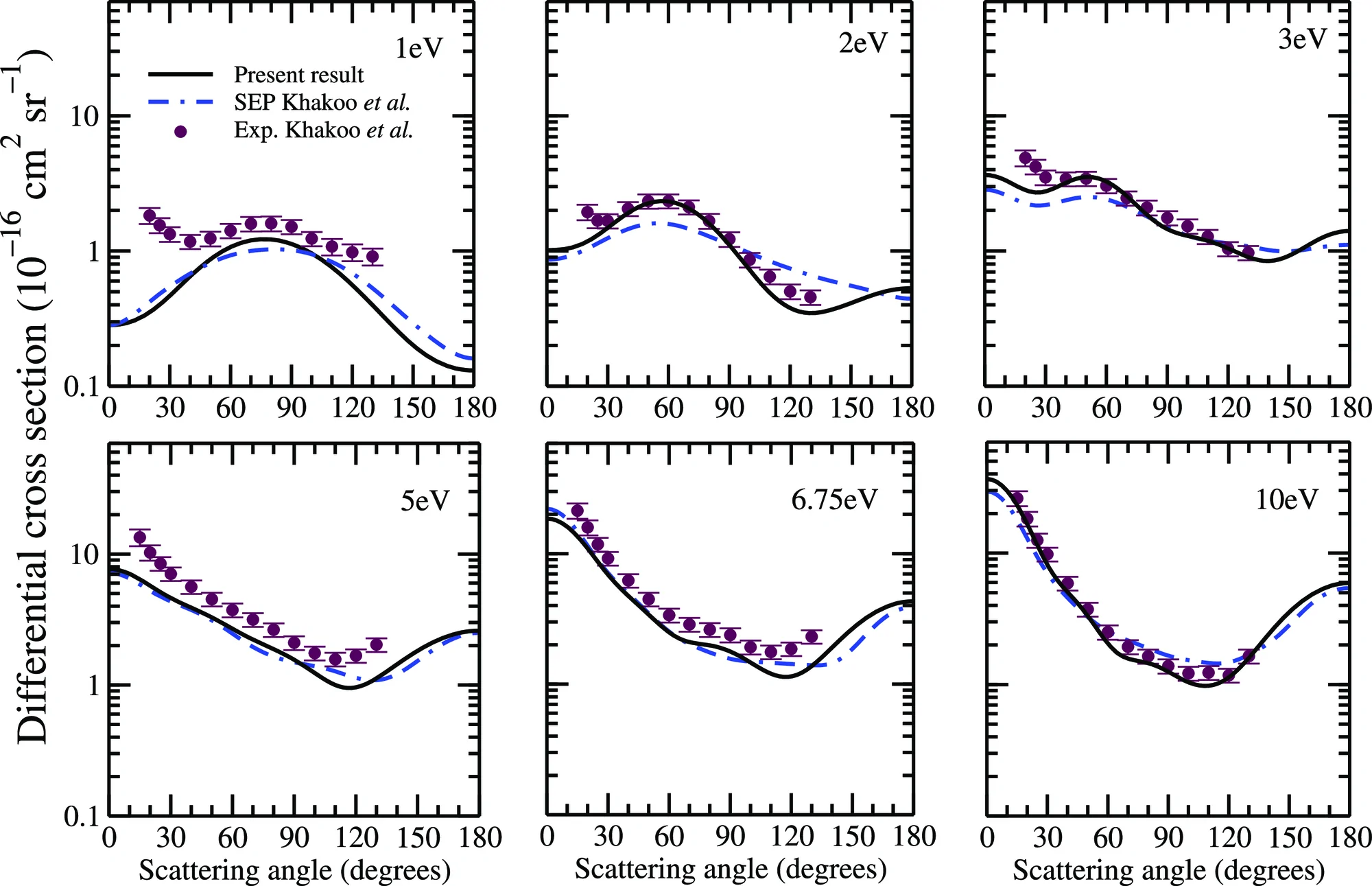
Differential cross section for elastic electron scattering by 2-butyne molecule at the impact energies of 1, 2, 3, 5, 6.75, and 10 eV. Solid black line, present result; dashed-dotted blue line, SMC results obtained by Khakoo et al.;^21^ maroon circles, experimental measurements performed by Khakoo et al.^21^

**Figure 6:**
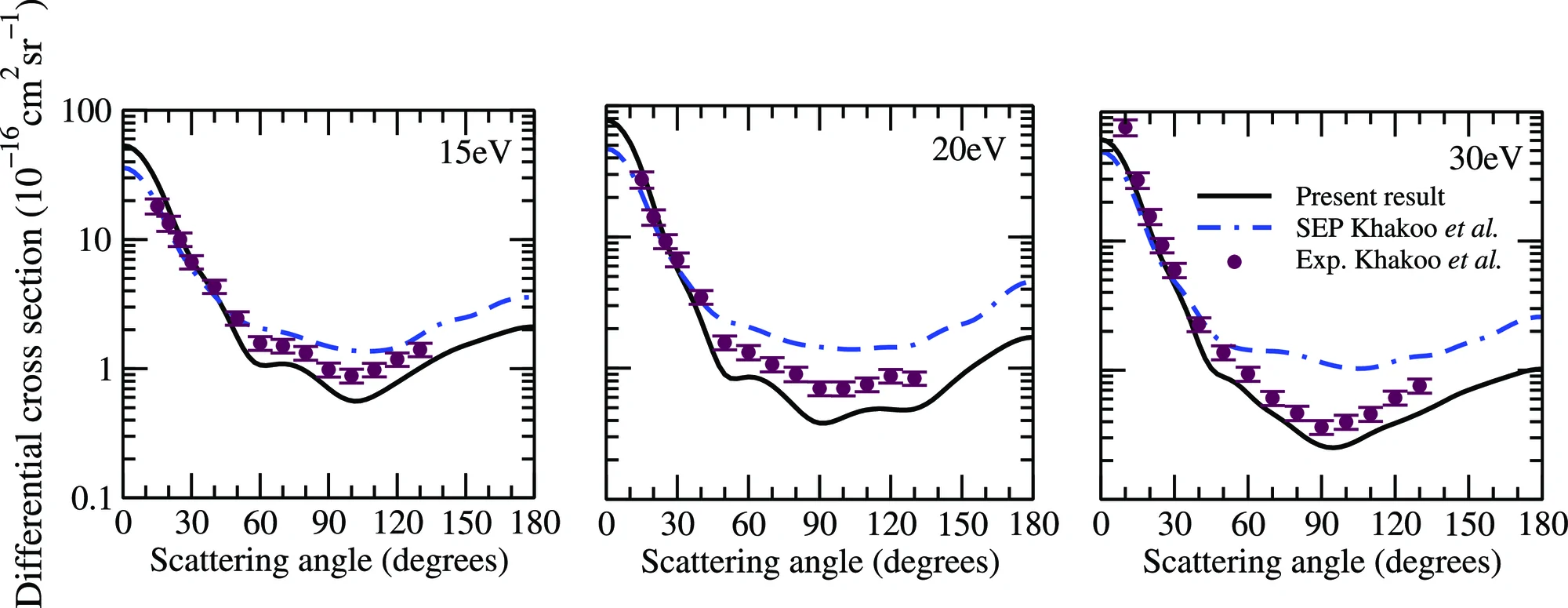
Differential cross section for elastic electron scattering by 2-butyne molecule at the impact energies of 15, 20, and 30 eV. Solid black line, present result; dashed-dotted blue line, SMC results obtained by Khakoo et al.;^21^ maroon circles, experimental measurements performed by Khakoo et al.^21^

### 4.3 Ionization Cross Section

The TICS for electron impact on the 2-butyne molecule was calculated using the BEB model, from the first ionization threshold (9.77 eV) up to 1000 eV, and is presented in Figure 7. The calculated value for the first ionization threshold is in excellent agreement with the experimental value reported by Carlier et al.^56^ (9.562 eV). No results for the ionization cross section of this system were found in the literature to date. As expected, the cross section exhibits a maximum around 70 eV, above which it decreases with increasing impact energy. Despite differences in magnitude, the TICS estimated in this work using the BEB model can be considered sufficiently reliable to be included in the procedure adopted for determining the TCS in our analysis.

**Figure 7:**
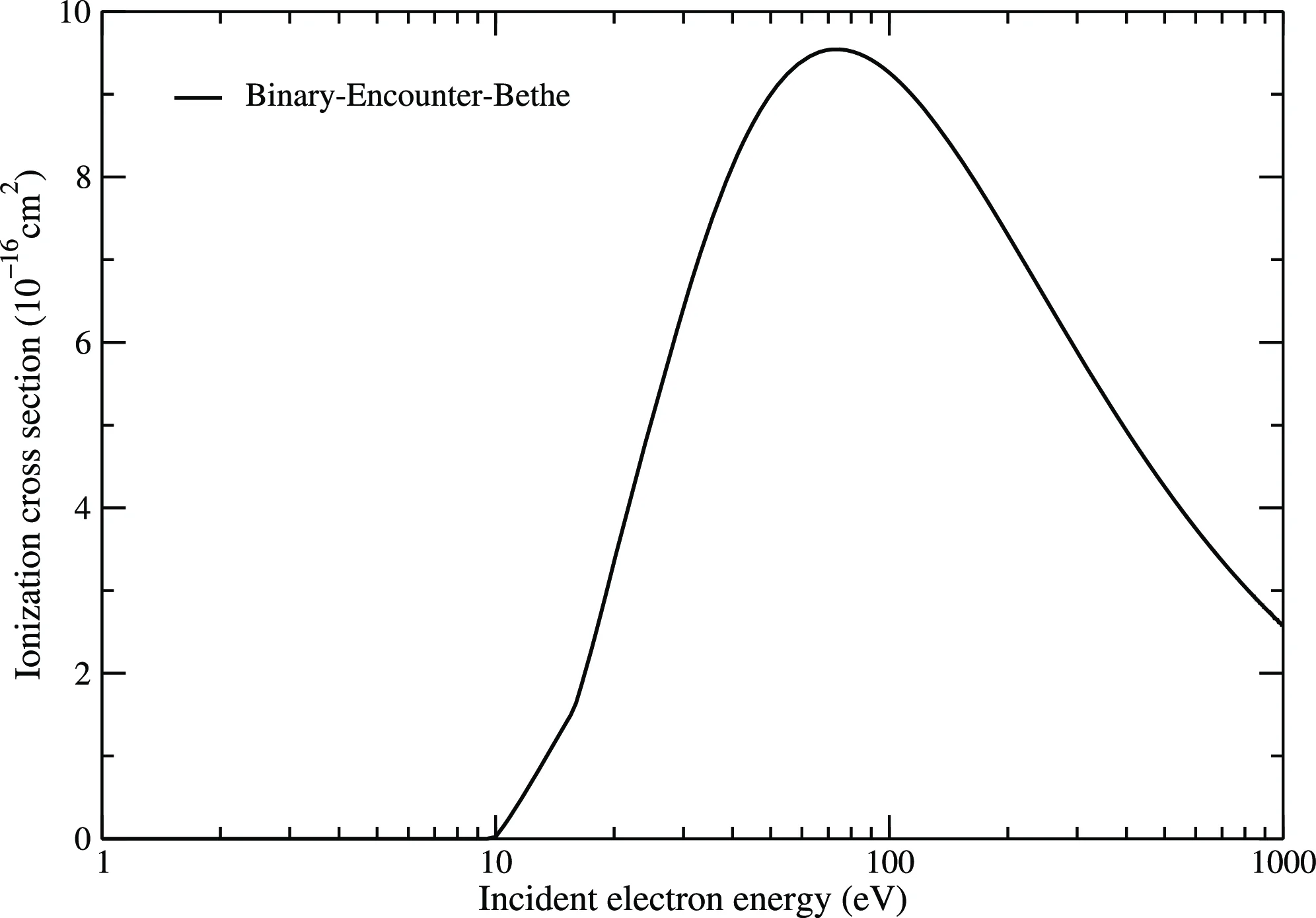
Ionization cross sections for electron scattering by the 2-butyne molecule. Solid black line, present BEB result.

### 4.4 Inelastic and Total Scattering Cross Sections

In Figure 8, we present the excitation cross sections from the ground state to the four lowest triplet states and the four lowest singlet states, namely 1: 1^3^ *B*_2_ (5.05 eV), 1^3^ *A*_2_ (5.76 eV), 2^3^ *B*_2_ (5.76 eV), 2^3^ *A*_2_ (6.45 eV), 1^1^ *A*_2_ (6.45 eV), 2^1^ *A*_2_ (6.79 eV), 1^1^ *B*_2_ (6.79 eV), 1^1^ *A*_1_ (6.93 eV) and 1^1^ *B*_1_ (6.93 eV) according C_2*v*_ point group, for impact energies ranging from 5 to 50 eV. For simplicity, we adopt the labels 1T, 2T, 3T, and 4T for the triplet states, and 1S, 2S, 3S, and 4S for the singlet states. To date, there are no results available in the literature that allow for a direct comparison with the data reported here. Therefore, the following analysis focuses on the intrinsic features of the calculated cross sections. It is observed that, for energies below 10 eV, the cross sections exhibit a significant number of structures. These features are likely associated with threshold effects and the presence of resonances. A detailed characterization of these structures is challenging due to the low magnitude of the cross sections in this energy region, which makes them particularly sensitive to such effects. At energies around 7, 10, and 15 eV, a sharp decrease in the cross sections is observed, associated with significant variations in the number of open channels. Around 7 eV, the number of channels (the channel-coupling scheme) increases from 12ch to 32ch; near 10 eV, from 32ch to 143ch; and around 15 eV, from 143ch to 244ch. These abrupt changes indicate the opening of multiple inelastic channels, which redistributes the probability flux among the available channels and results in a reduction of the cross sections for specific transitions. Conversely, in the region around 20 eV, the observed structure is significantly less pronounced compared to those near the previous thresholds. This is due to the smaller variation in the number of open channels, increasing from 244ch to 247ch, which implies a much more subtle effect on the redistribution of the scattering flux. The 2T and 3T states are degenerate, which is directly reflected in the behavior of their cross sections, exhibiting very similar profiles. The same holds for the 2S and 3S states, which are also degenerate and display analogous behavior. In contrast, the 1S and 4T states have the same energy but different multiplicities. This energy coincidence characterizes an accidental degeneracy, since these states are not related by any symmetry of the system that enforces energy equality, which is reflected in the distinct behavior of the cross sections for both states. This coincidence may be associated with limitations of the employed methodology, such as the use of the HF level and the restriction to single excitations, which do not fully capture electron correlation effects and may lead to an inaccurate description of energy separations. Nevertheless, these aspects do not compromise the qualitative analysis of the results.

**Figure 8:**
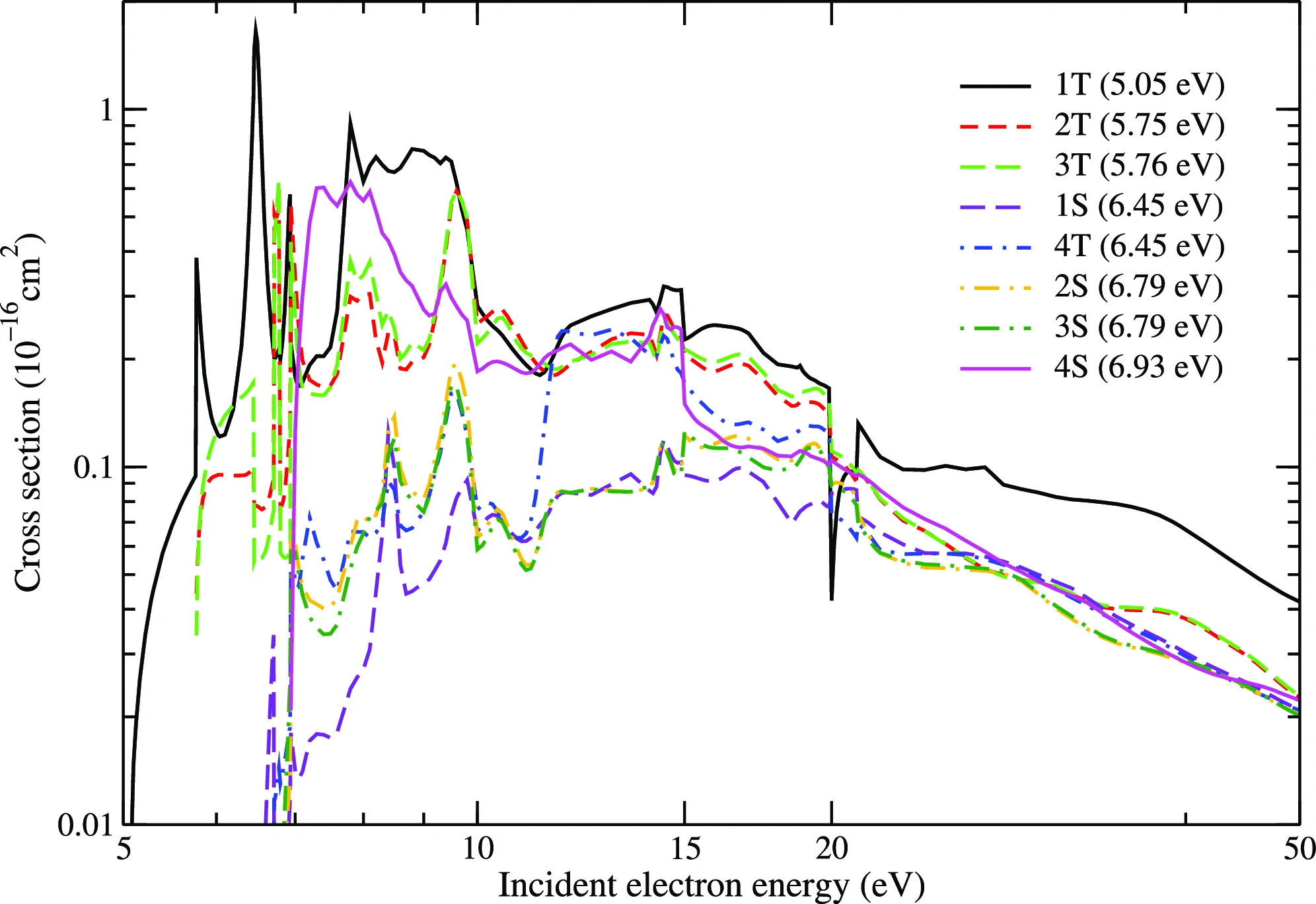
Integral cross sections for electronic transitions from the ground state to the first four triplet and singlet excited states of 2-butyne for electron-impact energies up to 50 eV. Solid black line is the present result for 1T state; dashed red line, for the 2T state; dashed green line, for the 3T state; dashed violet line, for the 1S state; dotted-dashed blue line, for 4T state; dotted-dashed yellow line, for the 2S state; dotted-dashed dark green line, for 3S state; and finally, solid magenta line is the present result for the 4S state.

In Figure 9, we present the differential cross sections (DCSs) for transitions from the ground state to the states 1T, 2T, 3T, 4T, 1S, 2S, 3S, and 4S, for impact energies of 10, 15, 20, 30, 40, and 50 eV. These energies were selected due to the presence of multiple structures at energies below 10 eV, which complicate a direct analysis.

**Figure 9:**
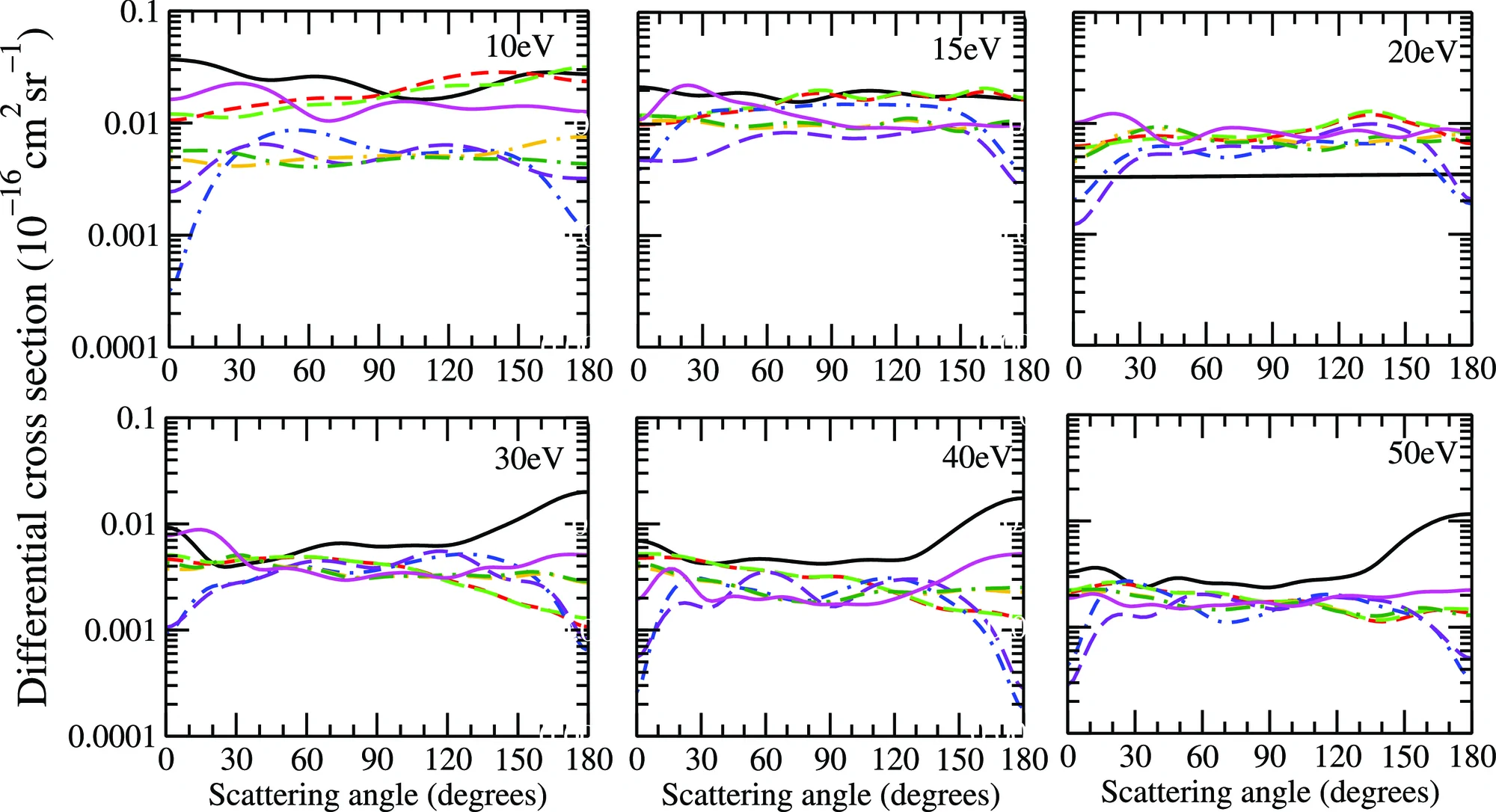
Differential cross sections for electronic transitions from the ground state to the first four triplet and singlet excited states of 2-butyne at selected impact energies of 10, 15, 20, 30, 40, and 50 eV. Solid black line is the present result for 1T state; dashed red line, for the 2T state; dashed green line, for the 3T state; dashed violet line, for the 1S state; dotted-dashed blue line, for 4T state; dotted-dashed yellow line, for the 2S state; dotted-dashed dark green line, for 3S state; and finally, solid magenta line is the present result for the 4S state.

For impact energies of 10 and 15 eV, the states 1T, 2T, 3T, and 4S exhibit larger magnitudes compared to the other states (1S, 2S, 3S, and 4T). This difference decreases at 20 eV, where all states display similar magnitudes. In particular, the DCS for the 1T state exhibits only a weak angular dependence, with a markedly reduced oscillatory pattern that results in an almost isotropic angular distribution. For energies above 30 eV, the 1T state exhibits the largest magnitude among all states, indicating a higher probability for this electronic transition of being accessed during the collision process, followed by the 2T and 3T states, and then the remaining states. The 4T and 1S states show a distinct behavior, with extremely reduced contributions to the cross section at 0° and 180°. Similar behaviors have been reported in the literature for other polyatomic systems. Cartwright et al.^57^ demonstrated that whenever the molecular target wave function changes parity during scattering, the DCSs vanishes exactly at these angles. This occurs because, in these geometries, the incident and scattered electron vectors lie within the same symmetry plane, so a transition between states of opposite parity would violate the reflection symmetry conservation of the total electron–target system. In our calculations for the 2-butyne molecule, analogous patterns are observed, particularly for transitions to the states 1^1^ *A*_2_ and 2^3^ *A*_2_. The ground state wave function 1^1^ *A*_1_ is symmetric with respect to both planes of the C_2*v*_ point group, whereas the wave functions of the excited states 1^1^ *A*_2_ and 2^3^ *A*_2_ are antisymmetric. Consequently, the transitions 1^1^ *A*_1_ → 1^1^ *A*_2_ and 1^1^ *A*_1_ → 2^3^ *A*_2_ are forbidden at scattering angles of 0° and 180°, resulting in DCSs that are close to zero in these directions. This effect is particularly pronounced for triplet transitions, while for singlet transitions the reduction is less severe, though still noticeable, which is consistent with what we observed in our results.

In Figure 10, we present the TCS, obtained as the sum of the elastic and electronically inelastic contributions calculated using the SMC method, together with the ionization contribution described by the BEB model, over the impact energy range from 0.1 to 50 eV. The electronically inelastic contribution corresponds to the sum of the excitation cross sections for the 247 target states explicitly included in the present MOB-SCI expansion, which covers excitation energies up to 21.06 eV. Therefore, the inelastic contribution shown in Figure 10 should be regarded as partial for impact energies above this threshold, since additional electronically excited states not included in the present expansion may become energetically accessible and contribute to the total electronically inelastic cross section. This approximate model has been widely employed in previous works by our group, showing good agreement with theoretical and experimental results available in the literature. In addition, we separately show the contributions of the elastic, inelastic and ionization channels to the TCS, as well as a comparison with experimental data reported by Szmytkowsi et al.^22^ for the total cross section. It is observed that, for energies below 10 eV, the TCS is dominated by the elastic channel. Above this energy range, the contributions from inelastic processes and ionization become progressively more significant, playing an important role in determining the TCS. Overall, a good agreement is observed between our results and the experimental data, highlighting the importance of including multiple scattering channels for a more complete and accurate description of the process.

**Figure 10:**
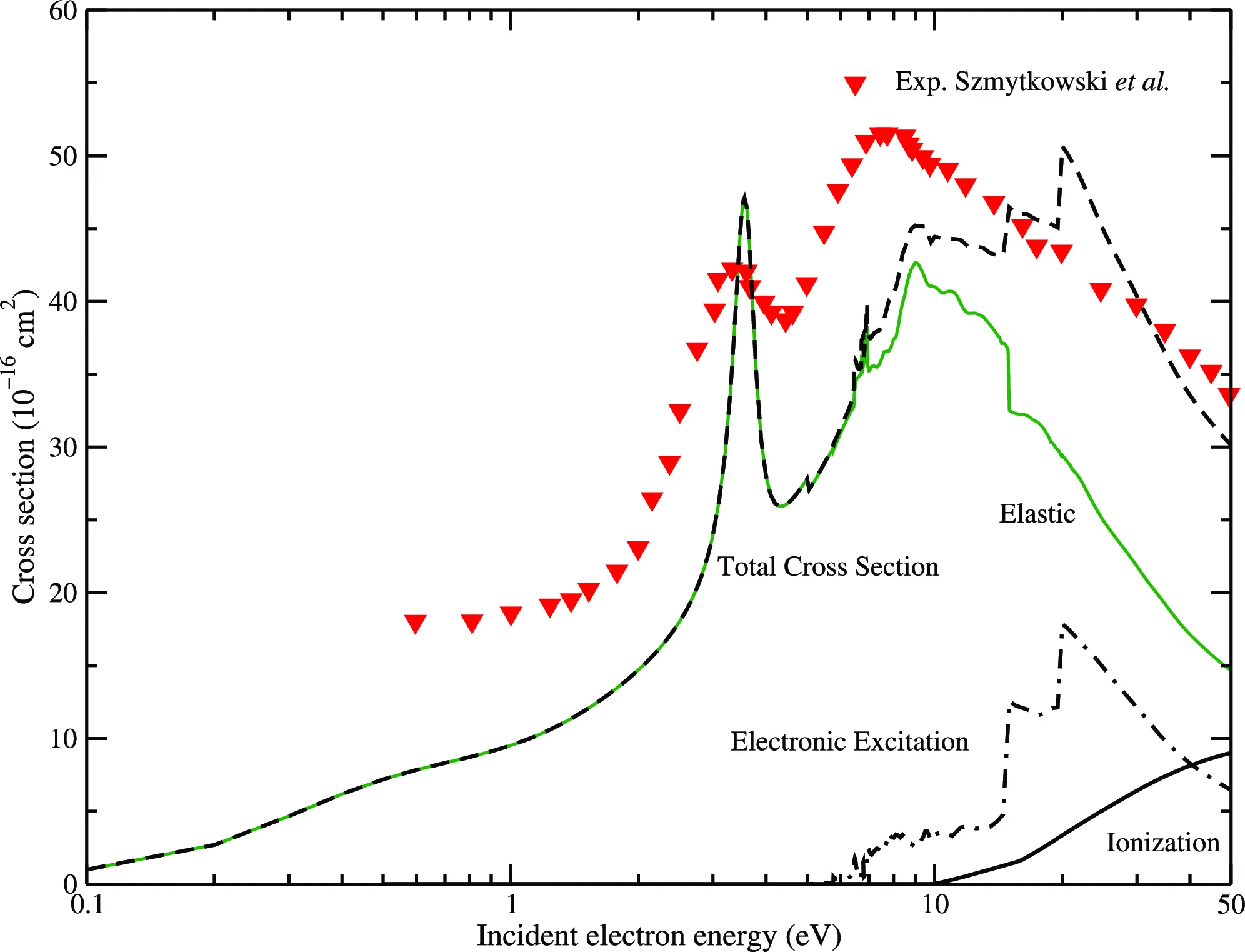
Cross section for electron scattering by the 2-butyne molecule. Full dark green line, present SMC ICS elastic; dashed black line, present SMC TCS elastic + inelastic + BEB result; dashed-dotted black line, present SMC inelastic result; full black line, ionization BEB result; and red triangles, TCS experimental measurements obtained by Szmytkowski et al.^22^

## 5 Conclusions

In this work, we present DCS and ICS for elastic and electronically inelastic electron scattering by the 2-butyne molecule, for impact energies ranging from 0.01 up to 50 eV, obtained using the SMC method. Our calculations include between 1 and 247 open channels, with electronically excited states described within the MOB–SCI strategy. We also report total ionization cross sections obtained using the BEB model. Based on these results, we estimate the TCS by summing the elastic and electronically inelastic contributions from the SMC calculations with the ionization contribution provided by the BEB model.

To the best of our knowledge, no results are available in the literature for the excited-state spectrum of the 2-butyne molecule. Therefore, additional calculations were performed using FSCI–PP (using pseudopotentials for calculation), FSCI–AE (including all target electrons), TD–DFT, and EOM–CCSD methods. Overall, a good agreement was obtained between the results derived from the MOB–SCI strategy and those from the other theoretical approaches. Due to the reduction of the molecular point group symmetry, some degenerate states, as well as accidental degeneracies, were observed. These features arise from methodological limitations; however, they do not affect the overall interpretation of the results. Regarding the elastic scattering calculations, good agreement was found with previous theoretical results and excellent agreement with the available experimental data. The differences in magnitude observed with respect to other theoretical studies can be attributed to the number of channels included in the calculations. While previous works considered only the elastic channel, our approach includes multiple open channels. This more comprehensive treatment of the scattering process leads to a significantly improved agreement with experimental measurements. In addition, the RT minimum previously reported in the literature was successfully identified in our results.

Regarding the inelastic results, no data are available in the literature for this scattering process. In this work, we present excitation cross sections from the ground state to the four lowest triplet states and the four lowest singlet states. For the ICS, in the low-energy region (below 10 eV), the small magnitude of the cross sections makes them particularly sensitive to threshold effects and resonances, which is reflected in the presence of several structures in this energy range. Above 10 eV, clear patterns emerge in the magnitude of the cross sections, possibly indicating differences in transition probabilities between electronic states. These features are more clearly analyzed in the DCSs. At 10 and 15 eV, the cross sections associated with the 1T, 2T, 3T, and 4S states exhibit larger magnitudes, followed by the 4T, 1S, 2S, and 3S states. For energies above 30 eV, the 1T state becomes dominant, followed by the 2T and 3T states, while the remaining states show smaller contributions. Additionally, the 4T and 1S states display a distinct behavior compared to the others at scattering angles of 0° and 180°, where the cross section tends to vanish. This behavior can be interpreted as a consequence of symmetry constraints, leading to a suppression of the scattering amplitude in these directions. Finally, as mentioned above, we estimate the TCS, obtaining good agreement between our results and the experimental data available in the literature.

Electronic excitation of molecular targets by electron impact remains a challenging scattering problem requiring a proper treatment of multichannel coupling. The Schwinger multichannel method has enabled predictive calculations for polyatomic targets. However, progress is severely constrained by the lack of comprehensive experimental data in the literature, particularly absolute cross sections for molecular excited states. We hope that this manuscript motivates new measurements.

## Data Availability

The data sets generated during and/or analyzed during the current study are available from the corresponding author on reasonable request.
